# Research on the Influence of the Type of Surfactant and Concentrator in Aqueous Dispersion of Pigments

**DOI:** 10.1007/s11743-013-1518-5

**Published:** 2013-08-18

**Authors:** Edwin Makarewicz, Agnieszka Michalik

**Affiliations:** Faculty of Chemical Engineering and Technology, University of Technology and Life Sciences, Seminaryjna St. 3, 85-326 Bydgoszcz, Poland

**Keywords:** Inorganic pigments, Water dispersion, Acrylic thickeners, Stability

## Abstract

This work reports tests performed to evaluate the stability of aqueous dispersions of inorganic oxide pigments with different specific surface areas, with the use of anionic and non-ionic surfactants and concentrators. Color mixtures of oxide compounds of blue, green, olive and brown with the unit cell spinel structure were used as pigments. The sodium salt of sulfosuccinic acid monoester, oxyethylenated nonylphenol and ethoxylated derivatives of lauryl alcohol, fatty alcohol and fatty amine were used as surfactants. The concentrators used were: poly(vinyl alcohol), the sodium salt of carboxymethyl cellulose as well as a water-based polyurethane oligomer. The highest dispersion efficiency was found for dispersed systems in which surfactant and concentrator were incorporated in the formula. The one containing the sodium salt of carboxymethyl cellulose or polyurethane oligomer with ethoxylated saturated fatty alcohol or fatty amine was found to be the most efficient. It was discovered that a higher dispersion efficiency corresponds to pigments with larger specific surface. The efficiency is also found to improve when the concentrator is an acrylic polymer or copolymer made up of two acrylic species. In this case, the concentrator interaction with the surfactant is more effective if the value of its boundary viscosity number is higher. This observation confirms the existence of interactions between macro-chains of the concentrator and surfactant molecules forming micelles with the pigment particles.

## Introduction

Water-based paints provide modern materials for painting. Together with pigments and fillers they make possible permanent dispersions of polymers in an aqueous environment. Surfactants are used for their stabilization as protective colloids and thickeners. They become adsorbed onto the surface of molecules of polymers, pigments and fillers until a state of balance is reached. Stability of a given dispersion system involves maintaining its properties unchanged throughout its volume, for an adequately long time [1–4].

Progress in the production technology of paints is connected with scientific research in the field of colloidal dispersion systems. It has enabled the development of a new theoretical basis for the construction of dispersion systems and also the development of stable compositions obtained by dispergation of pigments and fillers in resin solutions or in aqueous dispersions of polymers [5–9].

Dispersion systems are characterized by a strong development of the phase separation border, e.g., the surface of a pigment particle with the dispersion environment. Occurrence of significant free surface energy reduction which leads to self-induced processes of coalescence and coagulation of particles is connected with this phenomenon. This means that dispersion systems are basically unstable and the processes of aggregation of the dispersed phase particles occur sooner or later, followed by their sedimentation following the formation of sediment. One of the methods of experimental definition of the phenomena occurring in the dispersion system are observations and sedimentation tests [10–16].

The minimum free surface energy corresponds to the thermodynamic state of stable dispersion systems. Preparation of a dispersion requires the control of some of its properties, especially the concentration of the disperse phase, size of particles, stability and viscosity of the system. The first authors involved in the research into dispersion properties were of the opinion that the surface tension is a very important factor defining the stability and size of particles. Certainly, a low value of surface tension is preferable for dispersion [17]. However, changes which take place in the electrical double layer created on the surface of the particle dispersion are of greater importance. A electrical double layer favors the dispersion stability and first of all it prevents the coagulation of molecules [18–20]. Stabilizers such as surfactants can be characterized by the so-called hydrophilic-lipophilic balance (HLB). It is assumed that the concentration of surfactant on the phase separation surface and properties of the absorption layer depend on the number and kind of polar groups as well as the structure and length of the carbohydrate chains [21]. An approximate value of the optimal amount of stabilizer can be calculated with reference to the size of the phase separation surface, thickness of the layer, and critical concentration of surfactant molecules in the system. The stabilizing influence of the surfactant involves not only decreasing the surface tension at the interface, but also forming a structural barrier within the volume which will ensure dispersion stability [22, 23].

An aqueous dispersion of a pigment is an unstable system, because. during the connection of two particles. a decrease in the surface area occurs and in consequence their agglomeration. In order to prepare a stable dispersion, a definite stabilizer is needed. Four kinds of stabilizing additives can be distinguished. The least effective ones are simple, non-organic electrolytes. The fourth kind includes fine grain insoluble powders. The powder particles form a mono-layer rim stabilizing the system. The pigment particles stabilized in water by a nonionic surfactant or water-soluble polymers usually have a slight negative charge due to an excessive adsorption of anions from the water phase. The value of electrokinetic potential, as one of the factors determining aggregation stability of the pigment particles, has been neither explicitly explained nor justified so far [24–26].

Nowadays, it is impossible to characterize the stability of the disperse system by means of one simple method. The instability is the result of collision of particles caused by Brownian motion and convection leading to contact and agglomeration. Thus, the influence of surfactant structure on the dispersion stability is a crucial issue depending of three factors: a geometric barrier involving the preservation of the minimal distance between molecules of the stabilizer during the phases and a definite ratio between the polar and non-polar groups, an energy barrier defining the minimal deformation strength of the adsorption layer and a concentration barrier conditioning the presence of a saturated adsorption layer on the particle surface [9, 27–29].

It is often said that the presence of a electrical double layer is the factor determining the stability of dispersion molecules. However, this approach needs to be questioned as there is neither qualitative nor quantitative correlation with the disperse system properties. The electrical double layer determines only the adsorption layer thickness and partly the diffusion layer thickness and it apparently increases the size of dispersed particles [8]. High-molecular stabilizers should be classified into a separate group. This is caused by the fact that their structure on the surface is different from the one for low-molecular compounds. The amount of low-molecular surfactant necessary for stabilization of a unit surface are of dispersed particles is significantly higher than the amount of high-molecular stabilizer [17, 22]. The possibility of cooperation between surfactant molecules and the remaining components should also be accounted for. The choice of a given surfactant should depend on the kind of disperse system which defines the structure and interaction of the surfactant with the phases. So far there has been no satisfying, theoretical description of the considered phenomena. Therefore, taking into account all the factors affecting the persistence of the dispersed system, it is necessary to take into consideration the results of experimental tests while choosing the surfactant [17, 18, 21].

Sedimentary tests play a very important role in the research on dispersed systems properties. Undoubtedly, defining the relationship between the dispersed system structure with regard to intermolecular interactions is an especially difficult task. The kind of internal structure formed is determined by the type of contacts occurring between particles of the dispersed phase. Research on the operation mechanisms of surfactants is of key importance for direct contacts of particles between each other and has a large influence on all kinds of stability effects. They affect the formation kinetics of the network and its rheological properties. Despite efforts of many researchers, a theoretical description and analysis of the phenomena occurring during the formation of the disperse system, its behavior during a long-term storage or definition of the destabilization mechanism is still to very clear. The above mentioned fact was an inspiration for the authors to take up research on the considered issue. The subject of sedimentary tests are aqueous dispersions of pigments stabilized by different surfactants and concentrators. The observation of the phenomena occurring and their interpretation will make it possible to determine the stability conditions of these systems [4, 9, 22, 30, 31].

The main purpose was to determine the influence of different kinds of surfactants on the persistence of aqueous pigment dispersions. Nonionic and anionic surfactants, especially with different degrees of ethoxylation, and thereby different surface activity, were used as well as concentrators commonly applied in emulsion paints and synthesized from acrylic monomers. The research goal was to find the most stable dispersed systems and finding the relationship between structure parameters and properties of surfactants and the factors determining the stability of these systems.

## Experimental

### Materials

Unlimited pigments were products from Permedia SA Chemical Plant in Lublin. Purification of pigments from surface active agents was performed by extraction with *n*-butyl acetate an analytically pure (product from Polish Chemicals Reagents in Gliwice). Table 1 indicates the types of pigments and some selected physico-chemical properties. The presented names, symbols and structural chemical compositions and the type of elementary crystographic cell come from the producers. The elementary cell of the examined pigments has a spinel structure and molecule size of basic fraction, ranging from 2.5 to 5.0 μm. The specific surface was determined in a laboratory of the Institute of Protection Coatings Technology at the University of Technology and Life Sciences (UTL) in Bydgoszcz.

**Table 1:** Types and chemical properties of the inorganic pigments used in experiments

Type of pigment	Qualitative chemical composition	Specific surface S*10^3^, (m^2^/g)	Langmuir equation constant *b*, (cm^3^/mg)
Blue oxide PE-110	CoAl_2_O_4_	15.612	0.048
Green oxide PE-608	CoZnCrO_4_	22.473	0.028
Olive oxide PE-610	ZnFeAlCrO_4_	1.379	0.307
Brown oxide PE-780	ZnFeAlCrO_4_	2.244	1.999

Table 2 shows the basic properties of the monomers used for the synthesis of acrylic polymers and copolymers by the emulsion method.

**Table 2:** Acrylic monomers used in the experiments

Chemical unit	Abbreviation	Structural formula	*M*_CZ_ [g/mol]	Density [g/cm^3^]	Refractive index, *n*	Hildebrand parameter δ [MPa]^0,5^
Acrylic acid	AA	H_2_C=CHCOOH	72.06	1.0511	1.4224	20.32
Methacrylic acid	MAA	H_2_C=C(CH_3_)COOH	86.09	1.0150	1.4314	17.71
Acrylic acid amide	AAA	H_2_C=CHCONH_2_	71.08	–	–	24.19
Methyl acrylate	MA	H_2_C=CHCOOCH_3_	86.09	0.9560	1.3984	19.79
Ethyl acrylate	EA	H_2_C=CHCOOC_2_H_5_	100.11	0.9234	1.4068	18.97
Butyl acrylate	BA	H_2_C=CHCOO(CH_2_)_3_CH_3_	128.17	0.9078	1.4190	18.42
Methyl methacrylate	MMA	H_2_C=C(CH_3_)COOCH_3_	100.11	0.9430	1.4145	18.77
Ethyl methacrylate	MEA	H_2_C=C(CH_3_)COOC_2_H_5_	114.14	0.9135	1.4140	18.56

All the monomers are products of Aldrich. Ammonium persulfate, analytically pure produced by Polish Chemicals Reagents in Gliwice, was used as the initiator. The emulgator was a mixture of surfactants of the anion type: sodium alkylbenzenesulfonate (NaABS) with nonionic ethoxylated (10EO) lauryl alcohol (C_12_H_25_-EO_10_-H) according to a 1:1 weight ratio and concentration of 0,088 [g/cm³]. For stabilization of the aqueous disperse system of pigments nonionic and anionic surface active agents were used: ethoxylated lauryl alcohol abbreviated as C_12_H_25_EO_10_H (sold as Rokanol L-10 by PCC Exol), ethoxylated derivatives of unsaturated fatty alcohols (C_17_H_34_EO_18_H sold as Rokanol O-18 and C_19_H_38_EO_20_H sold as Rokanol K-20 by PCC Exol), ethoxylated tallow amine (C_17_H_35_NHEO_22_H sold as Rokamin SR-22 by PCC Exol) and disodium salt of sulfosuccinic acid monoester of ethoxylated (5 EO group) nonyl phenol by PCC Exol (C_9_H_19_C_6_H_4_OEO_5_COCH_2_CH_2_CO abbreviated as Sulfosuccinate N5 in what follows). Table 3 shows their characteristics and basic properties in water solutions.

**Table 3:** Properties of used surfactants

Surfactant	Molecular weight* M*_cz_ (g/mol)	HLB	CMC 10^3^ (mol/dm^3^)	Surface activity, G 10^−3^ (mN/m × dm^3^/mol)	Critical viscosity number GLL (cm^3^/g)	Haller interaction constant, k	Hildebrand solubility parameter δ (MPa)^1/2^
C_12_H_25_EO_10_H	610	14.4	0.80	47.8	0.0013	0.17	17.14
C_17_H_34_EO_18_OH	1013	15.6	0.30	74.3	0.0017	0.17	12.44
C_19_H_38_EO_20_OH	1163	15.1	0.30	84.1	0.0036	0.16	16.73
C_17_H_35_NHEO_22_H	1227	15.8	0.08	68.3	0.0016	0.18	13.26
Sulfosuccinate N5	648	10.0	0.06	53.7	0.0017	0.24	14.48
NaABS	354	5.8	0.02	51.7	0.0011	0.12	16.73

The chemical names and molecular masses of the surfactants come from the producer, whereas the remaining properties were determined or calculated by the authors of this paper. All surfactants were products of PCC Rokita SA in Brzeg Dolny. Apart from the synthesized acrylic polymers and copolymers, several other compounds were used as concentrators, namely: poly(vinyl alcohol) (PVA) Polyviol-G 26/140, a product of Wacker-Chemie (Germany), sodium salt of carboxymethyl cellulose (NaKMC) of high viscosity with 1 % weight of water solution, in 25 [°C], 1,5-3,0 [Pa·s], product of Chemical Co. (USA) and water-based polyurethane oligomer (PU) Schwego PUR 8050 with 15 % mass concentration, product of Schwegomann (Germany). For comparative purposes, Concentrator T was used, being an aqueous dispersion of acrylic copolymer stabilized by a mixture of anionic and nonionic surfactants with pH 7.5, product of the Institute of Polymer Materials Engineering and Paints in Toruń, Department of Rubber and Vinyl in Oświęcim.

### Research Methodology

Pigments were purified through extraction by *n*-butyl acetate in a Soxhlet apparatus for 2.5 h, then they were dried in a thermostat at about 100 °C until a solid mass was attained. In this way, the modifier adsorbed onto the surface of the pigments, i.e. the surfactant—oleic acid, was removed. The Hildebrand solubility parameter of the surfactant was calculated on the basis of data given in tables presented elsewhere [26]. The value of the hydrophilic-lyophilic balance (HLB) was calculated according to the formula reported in the literature [16–18]. The critical micelle concentration (CMC) was determined by an experimental method, on the basis of the dependence between the surface tension measured with a Du Nouy tensiometer and surfactant aqueous concentration [17, 18]. The CMC value corresponds to the graphically obtained minimal value of the surface tension. Surface activity (G) of the surfactant in water was calculated on the basis of the Rebinder formula reported in the paper [23]. The boundary viscosity number of the surfactant (GLL_SAA_) solutions and concentrators was determined from viscosity measurements performed using a Ubbelhod capillary viscometer. Calculations were carried out using Haller and Huggins equations [27]. The percentage content of surfactant or concentrator was calculated from the ratio of the mass after drying the sample on a Petri dish at 100 °C for 48 h, to the weighed mass of surfactant or the concentrator.

### Methods of Emulsion Polymerization and Copolymerization Performance

The laboratory apparatus consisted of a three-neck flask of 1000 cm³ capacity, equipped with a reflux condenser and connected to an absorber filled with silicone gel, a mechanical stirrer with a rotation regulator, a control thermometer up to 100 °C, a connection to gaseous nitrogen, a dropper of 200 cm^3^ capacity, a bubbler for distillation with water vapor, a distillation head and a bath with a temperature regulator for the use of a contact thermometer. First, 400 cm³ of water with 2.5 cm³ monomers and 2.5 cm³ emulsifier were put into the reaction flask. The amount of the initiator was constant and equal to 0.55 g (0.0024 mol). Then, 1.5 mol of acrylic amide, poly(acrylic acid) and poly(methacrylic acid) and 2 mol of acrylic acid or methacrylic acid were used for the synthesis of poly(acrylic amide). For the synthesis of copolymers consisting of acrylic or methacrylic acid esters, 2.0 mol of these compounds were used. The second co-monomer was acrylic or methacrylic acid in fixed quantity equal to 0.16 mol. The components were stirred and heated. Then, the temperature was gradually raised to 80–85 °C for 1 h. During this time, starting from 60 °C, an initiator was added in small portions. After 4 h of reaction at 80–85 °C, the synthesis of the polymer was terminated. The unprocessed monomer was removed through distillation using water vapor. The latex, cooled and filtered through a cotton baffle, was used for the tests. A 10 % solution of NaOH was used for pH correction to within the range of 7.2–7.5. After neutralization, the aqueous solution of acrylic concentrator was ready for further tests.

### Viscosity Characteristics of Synthesized Polymers and Copolymers

**Fig. 1:**
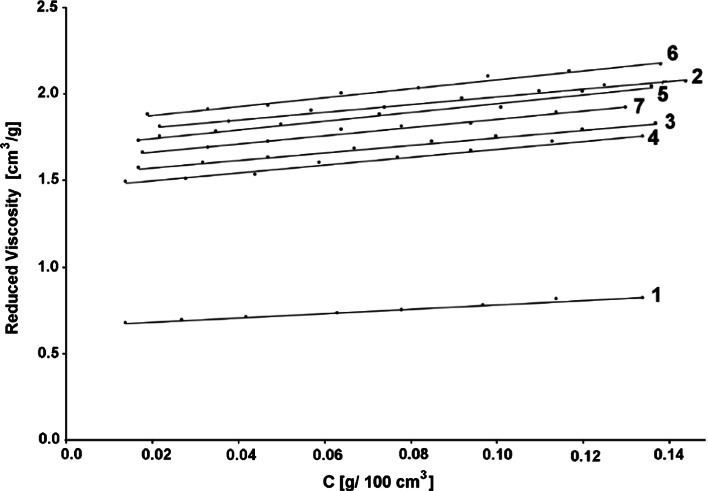
Reduced viscosity versus polymer concentration in the water. Explanation: *1* PVA, *2* NaCMC, *3* PU, *4* Thickener T, *5* PAA, *6* PMAA, *7* PAAA. The full names of the thickeners are shown in Table 4

**Fig. 2:**
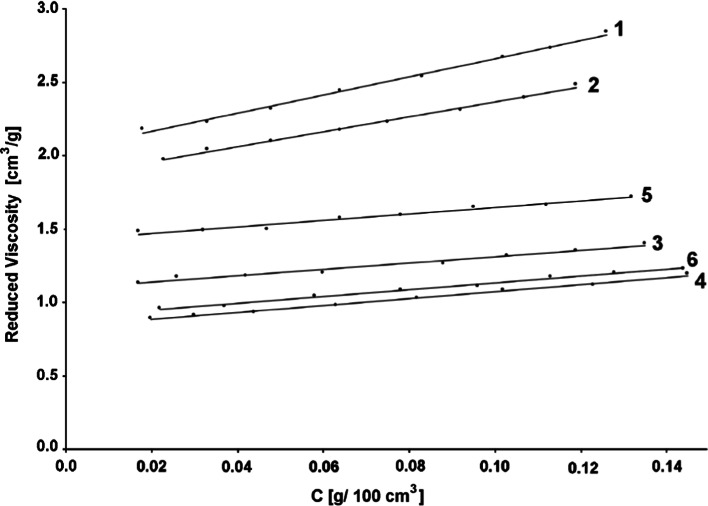
Reduced viscosity versus polymer concentration in the water. Explanation: *1* MA/AA, *2* BA/AA, *3* MMA/AA, *4* MEA/AA, *5* EA/MAA, *6* MEA/MAA. The full names of the thickeners are shown in Table 4

Figures 1 and 2 show the results of viscosity tests for all the synthesized concentrators, as well as those used for tests, as the reduced viscosity versus the polymer aqueous concentration. Concentrations of the polymer in water were matched experimentally so that the tested systems would exhibit a Newtonian flow.

The calculated values of the boundary viscosity number and Huggins constant are indicated in Table 4.

**Table 4:** Boundary viscosity number and Huggins constant for used thickeners

Thickener	Designation	Boundary viscosity number (cm^3^/g)	Huggins constant
Poly(vinyl alcohol)	PVA	0.6346	3.637
Carboxymethyl cellulose sodium salt	NaCMC	1.7628	0.713
Water-soluble polyurethane oligomer	PU	1.5316	0.928
Acrylic copolymer	Thickener T	1.4497	1.101
Poly(acrylic acid)	PAA	1.6898	0.893
Poly(methacrylate acid)	PMAA	1.8264	0.771
Poly(acrylate amid)	PAAA	1.6183	0.901
Methyl acrylate–acrylic acid copolymer	MA/AA	2.0402	1.485
Butyl acrylate–acrylic acid copolymer	BA/AA	1.8562	1.470
Methyl methacrylate–acrylic acid copolymer	MMA/AA	1.0911	1.824
Ethyl methacrylate–acrylic acid copolymer	MEA/AA	0.8343	3.435
Ethyl acrylate–methacrylic acid copolymer	EA/MAA	1.4233	1.089
Ethyl methacrylate–methacrylic acid copolymer	MEA/MAA	0.8988	2.874

### Method of Pigment Aqueous Dispersion Preparation

Measured quantities, i.e. 20.0 g, of pigment, surfactant or concentrator of the first or the second component were put into the homogenizer which was next filled with distilled water up to 50 cm³. Dispersion was performed by means of a laboratory homogenizer of the Ika-Ultraturrax T-25 type with a mixer of the Ika-S25 N-18 g type, for 20 min. Aqueous dispersions of pigments were subjected to sedimentation tests. Sedimentation measurements were performed in cylinders with 50 cm³ capacity [31]. They enabled us to determine the so-called dispersion efficiency (W_d_,  %) which reflects the ratio of the pigment quantity of the aqueous dispersion stable phase to the quantity of pigment used for preparation of the test. Ten samples containing different quantities of pigment were prepared for one pigment and one stabilizing system [7–9].

## Results and Discussion

**Fig. 3:**
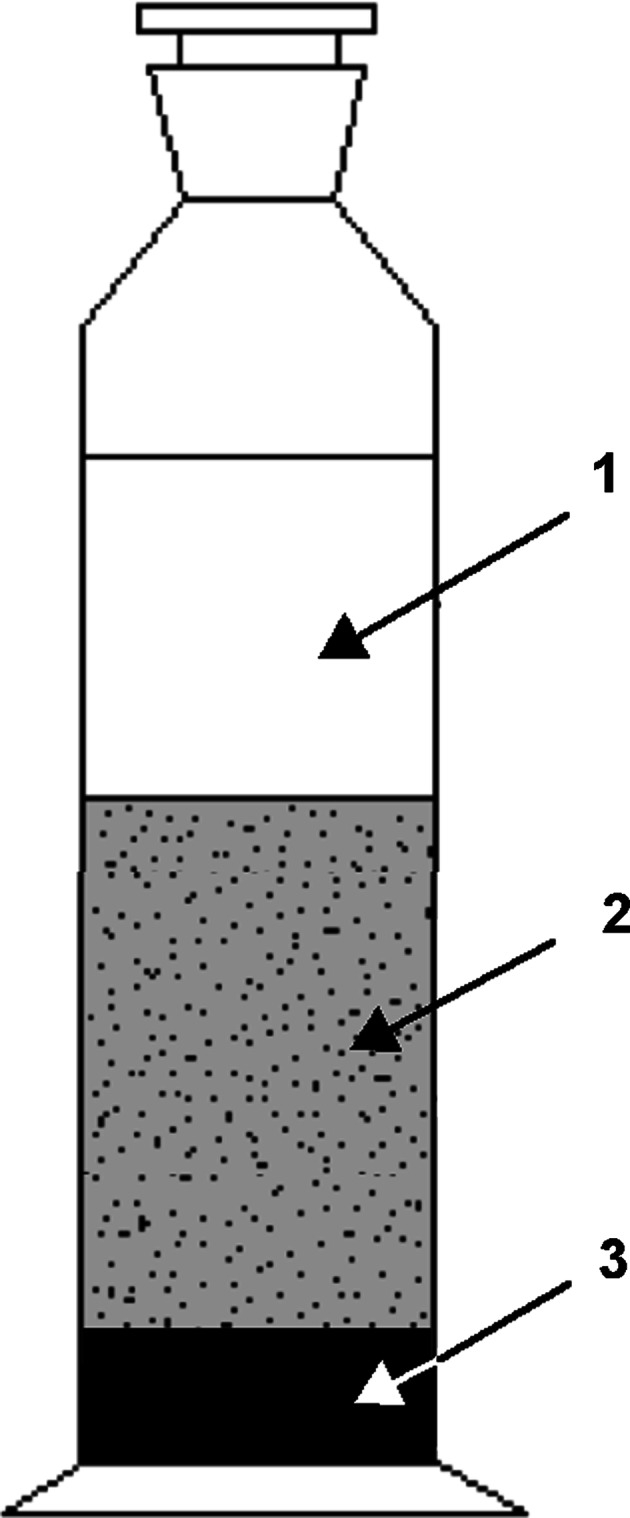
Schematic representation of the sedimentation studies conducted in cylinder. Explanation: *1* water phase, *2* stable phase of pigment dispersion in water, *3* sediment layer of pigment

The dispersion of pigments in water with surfactants and concentrators results in a stable dispersion system. The adsorption layers formed on the surface of the pigment particles by the surfactant or concentrator provide the system with some stability against aggregation and sedimentation. It is affected by the flocculation and agglomeration of the pigment micellar particles occurring inside the dispersed system volume, leading to formation of a separate water phase and pigment sedimentation. The speed of separation of the pigment dispersion into separate phases depends on the difference in density, the viscosity of the aqueous phase and the size of the pigment particles. After a given time, a state of sedimentation equilibrium is reached by the system which does not exhibit any more alteration. The results of the sedimentation tests performed in cylinders are presented in Fig. 3.

**Fig. 4:**
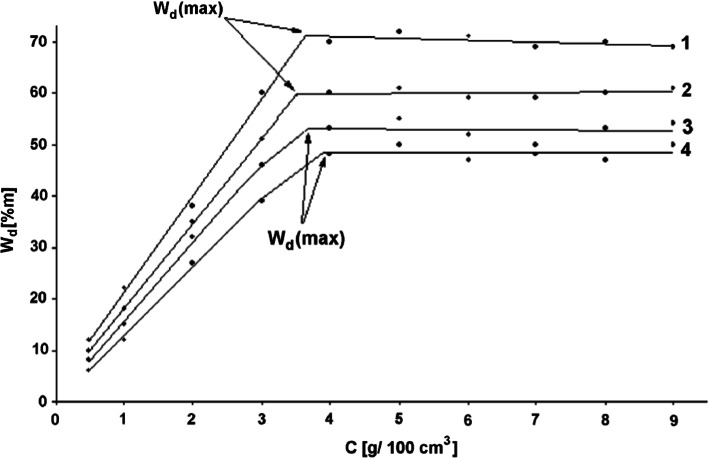
Efficiency of pigment dispersion versus (C_12_H_25_EO_10_) surfactant concentration (C_S_) in water. Explanation: *1* Green oxide PE-608, *2* Blue oxide PE-110, *3* Brown oxide PE-780, *4* Olive oxide PE-610

In fact, after 2 days of the sedimentation exposition in the disperse system, a sedimentation equilibrium was reached. In order to determine the dependence between the pigment dispersion efficiency and the concentration of the surfactant or concentrator, it was necessary to perform 10 tests for different conditions. This provided the basis for the determination of the maximal aqueous dispersion efficiency of a pigment, and accordingly, the optimal surfactant concentration value. These tests were also conducted for all the pigments and formulations of stabilizers containing surfactants and concentrators. Figure 4 shows the results of the sedimentation tests of aqueous dispersions of pigments by means of C_12_EO_10_.

On the sedimentation curves, presented in Fig. 4, the maximal value of pigment aqueous dispersion efficiency was marked as W_d(max)_. Tests for the remaining surfactants, concentrators and their combinations were performed in an analogical way. Table 5 shows the determined values of the maximal dispersion efficiency and corresponding values of surfactant optimal concentration.

**Table 5:** Maximum value of dispersion efficiency and optimum content of surfactant

Pigment name and symbol	C_12_H_25_EO_10_		C_17_H_34_EO_18_OH		C_19_H_38_EO_20_OH		C_17_H_35_NHEO_22_H	
	*W*_d(max)_ (%)	*C*_S _(g/dm^3^)	W_d(max)_ (%)	*C*_S_ (g/dm^3^)	*W*_d(max)_ (%)	*C*_S_ (g/dm^3^)	*W*_d(max)_ (%)	*C*_S_ (g/dm^3^)
Green oxide PE-608	70.0	3.5	80.0	3.0	80.0	3.0	85.0	3.5
Blue oxide PE-110	60.0	3.5	65.0	3.0	73.0	3.5	81.0	3.5
Brown oxide PE-780	53.0	4.0	55.0	4.0	57.0	4.0	74.0	4.0
Olive oxide PE-610	48.0	4.0	50.0	4.0	52.0	4.0	55.0	4.0

*C*_*S*_ optimum concentration of surfactant

The results presented in Table 5 unequivocally indicate that the value of dispersion efficiency rises with the increase in pigment specific surface area. An increase in the nonionic surfactant ethoxylation also contributes to improving the dispersion efficiency. It has been found that the maximal values of dispersion efficiency are reached for similar quantities of the surfactant. Slightly higher amounts of surfactants are required for systems containing pigments with a small specific surface area. In this case, the most efficient surfactants appeared to be C_17_H_34_EO_18_OH and C_17_H_35_NHEO_22_H.

Successive tests involved defining the influence of the type and quantity of concentrator, that is poly(vinyl alcohol), sodium salt of carboxymethyl cellulose and water-based polyurethane oligomer on the value of pigment aqueous dispergation efficiency. In Table 6 the results of the tests performed are presented.

**Table 6:** Maximum value of dispersion efficiency and optimum content of thickener

Pigment name and symbol	Poly(vinyl alcohol), PVA		Sodium salt of carboxymethyl cellulose, NaCMC		Water-based polyurethane oligomer, PU	
	*W*_d(max)_ (%)	*c*_thic._ (g/dm^3^)	*W*_d(max)_ (%)	*c*_thic._ (g/dm^3^)	*W*_d(max)_ (%)	*c*_thic._ (g/dm^3^)
Green oxide PE-608	20.0	20.0	100.0	2.0	100.0	3.5
Blue oxide PE-110	20.0	20.0	100.0	2.0	100.0	3.5
Brown oxide PE-780	5.0	20.0	100.0	2.5	100.0	5.5
Olive oxide PE-610	5.0	20.0	100.0	2.5	100.0	6.0

Results presented in Table 6 prove that the most effective concentrator is the sodium salt of carboxymethyl cellulose and the second best is the water-based polyurethane oligomer. Poly(vinyl alcohol) turned out to be the least efficient concentrator. It can be said that in this case, the attainment of a stable aqueous dispersion of pigment is caused by increased viscosity of the dispersing environment. The value of viscosity is such that practically no particle settling takes place. Consistency of the dispersion environment is similar to non-Newtonian viscous-plastic liquids (jelly like). Dilution of the system with water leads to sedimentation of the pigment and formation of sediment in the cylinder bottom. Disperse systems with such properties cannot be applied to the production of paints. Only those dispersed systems which may be diluted without a significant change in stability, find a practical application.

For further tests, aimed at defining stability of pigment disperses systems, a formula consisting of a nonionic surfactant and a concentrator was used. Table 7 shows the results of different tests.

**Table 7:** Maximum value of dispersion efficiency and optimum content of thickener and surfactant for different pigments

Pigments name and symbol	PVA–C_12_H_25_EO_10_			PVA–C_17_H_34_EO_18_OH			PVA–C_17_H_35_NHEO_22_H		
	*W*_d(max)_ (%)	*c*_thic._ (g/dm^3^)	*C*_S_ (g/dm^3^)	*W*_d(max)_ (%)	*c*_thic._ (g/dm^3^)	*C*_SA_ (g/dm^3^)	*W*_d(max)_ (%)	*c*_thic._ (g/dm^3^)	*C*_S_ (g/dm^3^)
Green oxide PE-608	50.0	15.0	4.0	80.0	15.0	2.0	80.0	12.0	3.0
Blue oxide PE-110	45.0	15.0	4.0	70.0	15.0	2.0	70.0	12.0	3.0
Brown oxide PE-780	25.0	20.0	10.0	62.0	20.0	4.0	55.0	20.0	4.0
Olive oxide PE-610	25.0	20.0	10.0	55.0	20.0	4.0	50.0	20.0	4.0

Pigments name and symbol	NaCMC–C_12_H_25_EO_10_			NaCMC–C_17_H_34_EO_18_OH			NaCMC–C_17_H_35_NHEO_22_H		
	*W*_d(max)_ %	*c*_thic._ (g/dm^3^)	*C*_S_ (g/dm^3^)	*W*_d(max)_ %	*c*_thic._ (g/dm^3^)	*C*_S_ (g/dm^3^)	*W*_d(max)_ %	*c*_thic._ (g/dm^3^)	*C*_S_ (g/dm^3^)
Green oxide PE-608	80.0	1.5	4.0	100.0	1.0	2.0	100.0	1.0	2.0
Blue oxide PE-110	80.0	1.5	4.0	100.0	1.0	2.0	100.0	1.0	2.0
Brown oxide PE-780	40.0	2.5	6.0	100.0	1.5	3.0	100.0	1.5	4.0
Olive oxide PE-610	40.0	2.5	6.0	100.0	1.5	3.0	100.0	1.5	4.0

Pigments name and symbol	PU–C_12_H_25_EO_10_			PU–C_17_H_34_EO_18_OH			PU–C_17_H_35_NHEO_22_H		
	*W*_d(max)_ (%)	*c*_thic._ (g/dm^3^)	*C*_S_ (g/dm^3^)	*W*_d(max)_ (%)	*c*_thic._ (g/dm^3^)	*C*_S_ (g/dm^3^)	*W*_d(max)_ (%)	*c*_thic._ (g/dm^3^)	*C*_S_ (g/dm^3^)
Green oxide PE-608	75.0	3.0	6.0	100.0	2.0	2.0	100.0	4.0	2.0
Blue oxide PE-110	75.0	3.0	6.0	100.0	2.0	2.0	100.0	4.0	2.0
Brown oxide PE-780	45.0	4.0	6.0	100.0	4.0	4.0	100.0	6.0	5.0
Olive oxide PE-610	45.0	4.0	6.0	100.0	4.0	4.0	100.0	6.0	5.0

C_S_ optimal surfactant concentrate

Table 7 results show that the best stabilizing system contains the carboxymethyl cellulose sodium salt with C_17_H_34_EO_18_OH or C_17_H_35_NHEO_22_H. Similar stabilizing properties have been shown by a formula of water-based polyurethane oligomer and C_17_H_34_EO_18_OH or C_17_H_35_NHEO_22_H. In this case poly(vinyl alcohol) in combination with nonionic surfactants make up the disperse system with the lowest stability. On the basis of the conducted tests, it may ve said that the higher dispersion efficiency is associated with the larger pigments specific surface area and a lower amount of concentrator and surfactant. During these tests, no so-called jelly was observed in any system, as it was in the case of systems stabilized only by carboxymethyl cellulose sodium salt.

Further tests using polymers or copolymers as the ester from acrylic or methacrylic acid or monomers, gave the results in Table 8.

**Table 8:** Maximum value of dispersion efficiency and optimum content of acrylic thickener for different pigments

Pigments name and symbol	Polyacrylic acid (PAA)		Polymetacrylic acid (PMAA)		Polyacryloamide (PAAA)		Methyl acrylate – acrylic acid copolymer (MA/AA)	
	*W*_d(max)_, (%)	*c*_thic._, (g/dm^3^)	*W*_d(max)_, (%)	*c*_thic._, (g/dm^3^)	*W*_d(max)_, (%)	*c*_thic._, (g/dm^3^)	*W*_d(max)_, (%)	*c*_thic._, (g/dm^3^)
Green oxide PE-608	93.0	1.10	95.0	1.20	86.0	1.30	98.0	1.48
Blue oxide PE-110	85.0	1.25	90.0	1.35	84.0	1.30	98.0	1.80
Brown oxide PE-780	50.0	2.00	58.0	1.75	51.0	1.95	86.0	1.95
Olive oxide PE-610	41.0	2.35	52.0	2.10	45.0	2.20	82.0	2.0

Pigments name and symbol	Butyl acrylate–acrylic acid copolymer (BA/AA)		Methyl metacrylate–acrylic acid (MA/AA)		Ethyl metacrylate–acrylic acid copolymer (MEA/AA)		Ethyl acrylate–metacrylic acid copolymer (EA/MAA)	
	*W*_d(max)_, (%)	*c*_thic._, g/dm^3^	*W*_d(max)_, (%)	*c*_thic._, (g/dm^3^)	*W*_d(max)_, (%)	*c*_thic._, (g/dm^3^)	*W*_d(max)_, (%)	*c*_thic._, (g/dm^3^)
Green oxide PE-608	93.0	1.42	71.3	1.50	62.0	1.62	87.0	1.52
Blue oxide PE-110	90.0	1.50	61.6	1.55	56.0	1.74	81.0	1.70
Brown oxide PE-780	82.0	1.95	54.0	1.60	36.0	1.85	75.0	1.81
Olive oxide PE-610	76.0	2.0	52.0	1.95	31.0	1.90	62.0	1.95

Pigments name and symbol	Ethyl metacrylate–metacrylic acid (MEA/MAA)		Polyacryloamide (PAAA) + C_17_H_34_EO_18_OH c_spc_ = 2,0 g/dm^3^		Polyacryloamide (PAAA) + Sulfosuccinate N-5 c_spc_ = 1,8 g/dm^3^		Thickener T	
	*W*_d(max)_, (%)	*c*_thic._, (g/dm^3^)	*W*_d(max)_, (%)	*c*_thic._, (g/dm^3^)	*W*_d(max)_, (%)	*c*_thic._, (g/dm^3^)	*W*_d(max)_, (%)	*c*_thic._, (g/dm^3^)
Green oxide PE-608	73.0	1.80	96.0	1.60	92.0	1.50	93.0	1.45
Blue oxide PE-110	65.0	1.85	96.0	1.68	92.0	1.57	92.0	1.50
Brown oxide PE-780	52.0	1.90	85.0	1.70	83.0	1.62	87.0	1.68
Olive oxide PE-610	48.0	1.98	82.0	1.72	77.0	1.68	85.0	1.75

Table 8 data show that the efficiency of the aqueous dispersion of pigments depends on their specific surface area and the type of acrylic concentrator applied. In each case, the dispersion efficiency rises with an increase in the pigment specific surface area, whereas the best concentrators appear to be those polymers or copolymers which contain acrylic monomers. For polyacrylamide, which was tested with and without the addition of surfactant, it was found that addition of nonionic C_17_H_34_EO_18_OH or anionic Sulfosuccinate N-5 results in an improvement of dispersion efficiency. Results of dispersion efficiency tests obtained with the use of concentrators, being acrylic or methacrylic polymers and copolymers, are comparable with concentrator T commonly used for regulation of emulsion paints viscosity. An interesting observation is that the efficiency of an aqueous dispersion of pigments was higher for those stabilized by means of acrylic polymers or copolymers which are made up of two acrylic monomers.

The adsorption tests of methylene blue on purified pigment surfaces show that active adsorption centers with negative charges occurring on their surfaces, as the paint is a cationic compound and it dissociates with a positive charge. This accounts for the structure of micelles created from the pigment and anionic or nonionic surfactant. It can be said, that the micelles having adsorption layers formed from anionic surfactants are more durable and stable than those with layers from nonionic ones. Introducing a concentrator into the dispersion caused a significant increase in the disperse system viscosity. In the present case, raising the dispersion stability is caused by a slower particle settling. Electrostatic interaction between the concentrator functional groups and the pigment particle charged surface, adsorption in active places of the pigment of the concentrator macro-chain, is not excluded. Interaction between the concentrator macro-chains and surfactant adsorption layer, on the surface of the pigment particle, is not ruled out either. An analysis of the calculated values of the Hildebrand solubility parameter for surface active agents and acrylic and methacrylic monomers indicates their significant similarity. This means that the polymers or copolymers obtained are well solvated and water soluble compounds. Table 9 shows the maximal dispersion efficiency versus the value of the concentrator optimal concentration and its boundary value of viscosity number, for all the tested pigments.

**Table 9:** Dispersion efficiency versus pigments and thickeners

Pigment name and symbol	Polyvinyl alcohol (PVA) GLL = 0.6346	Sodium salt of carboxymethyl cellulose (NaCMC) GLL = 1.7628	Water-soluble polyurethane oligomer (PU) GLL = 1.5316
	W_d(max)_/c_thic._	W_d(max)_/c_thic._	W_d(max)_/c_thic._
Green oxide PE-608	1.00	50.00	28.60
Blue oxide PE-110	1.00	50.00	28.60
Brown oxide PE-780	0.25	40.00	18.20
Olive oxide PE-610	0.25	40.00	18.20

The results presented in Table 9 indicates that a higher value of the concentrator boundary viscosity number corresponds to a higher value of dispersion efficiency with a smaller concentration in the dispersion. On the basis of this data, it can be said that molecules of the concentrator whose boundary viscosity number is higher, occupy more water volume as they are more swollen and solvated by water dipoles. Thus, they increase the viscosity of the aqueous environment more effectively and can interact better with the pigment surface. This is one of the ways to explain the highest values of pigments dispersion efficiency in the presence of the carboxymethyl cellulose sodium salt.

However, when we consider an aqueous dispersed pigment system stabilized by a formulation consisting of a surfactant and a concentrator, then, the maximal value of the dispersion efficiency is divided by the total amount of surfactant and concentrator, and the results are shown in Table 10.

**Table 10:** Dispersion efficiency versos the amount of surfactant and thickener in the aqueous dispersion of pigments

Pigments name and symbol	PVA–C_12_H_25_EO_10_	PVA–C_17_H_34_EO_18_OH	PVA–C_17_H_35_NHEO_22_H
	*W*_d(max)_/(*c*_thic._ + *C*_S_)	*W*_d(max)_/(*c*_thic._ + *C*_S_)	*W*_d(max)_/(*c*_thic._ + *C*_S_)
Green oxide PE-608	2.63	4.88	5.33
Blue oxide PE-110	2.37	4.12	4.67
Brown oxide PE-780	0.83	2.58	2.29
Olive oxide PE-610	0.83	2.29	2.08

Pigments name and symbol	NaCMC–C_12_H_25_EO_10_	NaCMC–C_17_H_34_EO_18_OH	NaCMC–C_17_H_35_NHEO_22_H
	*W*_d(max)_/(*c*_thic._ + *C*_S_)	*W*_d(max)_/(*c*_thic._ + *C*_S_)	*W*_d(max)_/(*c*_thic._ + *C*_S_)
Green oxide PE-608	14.55	33.33	33.33
Blue oxide PE-110	14.55	33.33	33.33
Brown oxide PE-780	4.71	22.22	18.18
Olive oxide PE-610	4.71	22.22	18.18

Pigments name and symbol	PU–C_12_H_25_EO_10_	PU–C_17_H_34_EO_18_OH	PU–C_17_H_35_NHEO_22_H
	*W*_d(max)_/(*c*_thic._ + *C*_S_)	*W*_d(max)_/(*c*_thic._ + *C*_S_)	*W*_d(max)_/(*c*_thic._ + *C*_S_)
Green oxide PE-608	8.33	25.0	16.67
Blue oxide PE-110	8.33	25.0	16.67
Brown oxide PE-780	4.5	12.5	9.09
Olive oxide PE-610	4.5	12.5	9.09

The corresponding data shown in Table 10 unequivocally prove that carboxymethyl cellulose sodium salt is the best concentrator, whereas, water-based polyurethane oligomer is the second best one. For the formulation of a concentrator with a surfactant, a higher dispersion efficiency of pigments can be attained using a smaller amount of the concentrator.

The tests results can be accounted for in the following way. First, a surfactant adsorbs on the surface of pigments. Macro-chains of the concentrator interact with the surfactant adsorption layer and the more intensive it is, the higher its boundary viscosity number is. It was found, that C_17_H_34_EO_18_OH and C_17_H_35_NHEO_22_H with carboxylmethyl cellulose sodium salt are the surfactants with the highest stabilizing effect on aqueous dispersion of pigments.

The presented finding was to be confirmed by means of tests with the use of another kind of concentrator. For this purpose, acrylic polymers and copolymers were considered. Table 10 shows the values of dispersion efficiency with respect to the concentrator unit concentration.

The data presented in Table 11 shows that using acrylic polymer as a concentrator, e.g. poly(acrylic acid) or copolymer consisting of two acrylic monomers like methyl acrylate with acrylic acid or butyl acrylate with acrylic acid having the highest boundary values of viscosity number, provides the best dispersion efficiency. Application of a surface active agent reduces the amount of concentrator that is required and increases the persistence of the pigment dispersion system.

**Table 11:** Dispersion efficiency for the pigments aqueous dispersion thickened with acrylic polymers and copolymers

Pigments name and symbol	Polyacrylic acid (PAA)	Polymetacrylic acid (PMAA)	Polyacryloamide (PAAA)	Methyl acrylate–acrylic acid (MA/AA)
	*W*_d(max)_/*c*_thic._	*W*_d(max)_/*c*_thic._	*W*_d(max)_/*c*_thic._	*W*_d(max)_/*c*_thic._
Green oxide PE-608	84.55	79.17	66.15	66.22
Blue oxide PE-110	68.00	66.67	64.62	54.44
Brown oxide PE-780	25.00	33.14	26.15	44.10
Olive oxide PE-610	17.45	24.76	20.45	41.00

Pigments name and symbol	Butyl acrylate–acrylic acid copolymer (BA/AA)	Methyl metacrylate–acrylic acid copolymer (MMA/AA)	Ethyl metacrylate–acrylic acid copolymer (MEA/AA)	Ethyl acrylate–metacrylic acid copolymer (EA/MAA)
	*W*_d(max)_/*c*_thic._	*W*_d(max)_/*c*_thic._	*W*_d(max)_/*c*_thic._	*W*_d(max)_/*c*_thic._
Green oxide PE-608	65.49	47.33	38.27	57.24
Blue oxide PE-110	60.00	39.74	32.18	47.65
Brown oxide PE-780	42.05	33.75	19.45	41.44
Olive oxide PE-610	38.00	26.67	16.32	31.8

Pigments name and symbol	Ethyl metacrylate–metacrylic acid copolymer (MEA/MAA) *W*_d(max)_/*c*_thic._	Polyacryloamide (PAAA) + C_17_H_34_EO_18_OH c_spc_ = 2.0 g/dm^3^ *W*_d(max)_/(*c*_thic._ + *C*_S_)	Polyacryloamide (PAAA) + Sulfosuccinate N-5 c_spc_ = 1.8 g/dm^3^ *W*_d(max)_/(*c*_thic._ + *C*_S_)	Thickener T *W*_d(max)_/*c*_thic._
Green oxide PE-608	40.55	26.67	27.87	64.14
Blue oxide PE-110	35.14	26.09	27.29	61.33
Brown oxide PE-780	27.37	23.00	24.27	51.79
Olive oxide PE-610	24.22	22.04	22.13	48.57

## Conclusions

The tests performed have shown that the preparation of a stable aqueous dispersion of pigments is possible when a surfactant is an adsorber on the pigment particles. Anionic agents are better adsorbed on the pigment surface area than the nonionic ones. More surfactant is adsorbed onto the pigments with a larger specific surface and these systems reveal higher stabilization, that is a higher dispersion efficiency. Application of concentrators such as carboxymethyl cellulose sodium salt and polyurethane oligomer in a disperse system instead of a surfactant, improves the dispersion efficiency. Stability of the disperse system is provided by its high viscosity which practically inhibits settling of the pigment particles. Adsorption of the concentrator macro-chains on the surface of the pigment, especially on its active centers, cannot be excluded.

The most efficient formula of a surfactant/concentrator mixture was found to be the one containing carboxymethyl cellulose sodium phosphate or polyurethane oligomer with C_17_H_34_EO_18_OH or C_17_H_35_NHEO_22_H. In this case, though smaller amounts of the surfactant and concentrator are required, a better dispergation efficiency is attained. The earlier mentioned principle that pigments with higher specific surface area are characterized by higher dispersion efficiency is corroborated. The efficiency also increases when the concentrators are acrylic polymers or copolymers made up of two acrylic monomers.

When the interaction of a concentrator with the adsorption layer of the surfactant is more intensive, the higher is the value of its organic viscosity number, with a higher solvation and macro-molecule swelling. This phenomenon is probably related to the interactions between macro-chains of the concentrator and surfactant molecules in complex micelles.
